# A rare symplastic or bizarre leiomyoma of the scrotum: a case report and review of the literature

**DOI:** 10.1186/1757-1626-1-381

**Published:** 2008-12-09

**Authors:** Junaid Masood, Stelios Voulgaris, Peter Atkinson, Tom W Carr

**Affiliations:** 1 Department of Urology, Southend University Hospital, Essex, UK; 2 Department of Pathology, Southend University Hospital, Essex, UK

## Abstract

**Background:**

We present a case of a symplastic or "bizarre" leiomyoma of the scrotum. Isolated cases of leiomyomas have been reported arising from the renal pelvis, bladder, spermatic cord, epididymis, prostate as well as the glans penis. However such mesenchymal lesions of the scrotum are very rare.

**Case presentation:**

Macroscopically the tumour was a well-circumscribed grey-white lesion 8.5 cm in size. Because of its peculiar histological characteristics this tumour was assigned as a symplastic or bizarre leiomyoma of the scrotum.

**Conclusion:**

We present this unusual tumour and highlight some important diagnostic and treatment pitfalls related to this rare tumour. This case demonstrates that leiomyomas should be considered in the differential diagnosis of scrotal tumours.

## Case presentation

**Figure 1:**
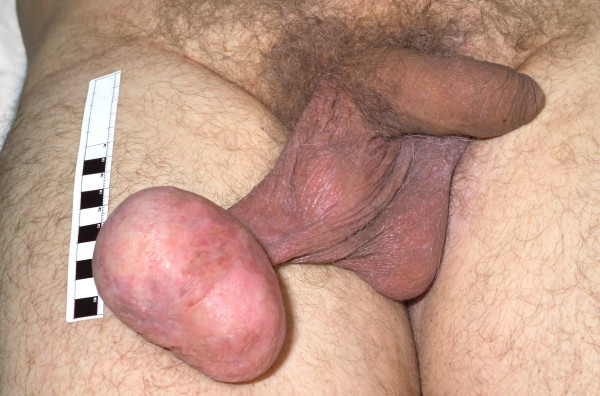
**Demonstrates the clinical appearance of this tumour arising from the dependant part of the right hemiscrotum**.

A 59-year old Caucasian man of average height and build presented with an 18-year history of a slowly enlarging, painless hard mass arising from the lower pole of his right scrotum (figure 1). There was no significant family or past medical history. On examination an 8 to 9 centimetre (cm) pedunculated, firm but smooth, non-tender mass was found arising from the dependant part of his right scrotum. This mass was not adhered to deeper layers and was not transilluminable.

A scrotal ultrasound confirmed normal testes and cord structures but showed a bizarre solid mass with a heterogeneous disorganised pattern with poor vascularity suggesting a mesenchymal tumour arising from the scrotal wall. This mass was excised under a general anaesthetic. The patient made a good recovery post-operatively. Macroscopically the tumour was a well-circumscribed grey-white lesion 8.5 cm in size. Because of its peculiar histological characteristics this tumour was assigned as a symplastic or bizarre leiomyoma of the scrotum.

## Discussion

Conventional leiomyomas may originate from any anatomic location of smooth muscle in the genitourinary system[1]. Isolated cases of leiomyomas have been reported arising from the renal pelvis, bladder, spermatic cord, epididymis, prostate as well as the glans penis [1-4]. However mesenchymal lesions of the scrotum are rare lesions[5]. Leiomyomas are well known to be by far the commonest neoplasm arising from the uterus[6].

Symplastic leiomyomas are rarely reported lesions in the medical literature [7-12] A medline search reveals less than 10 reports of symplastic or "bizarre" leiomyoma of the scrotum.

**Figure 2:**
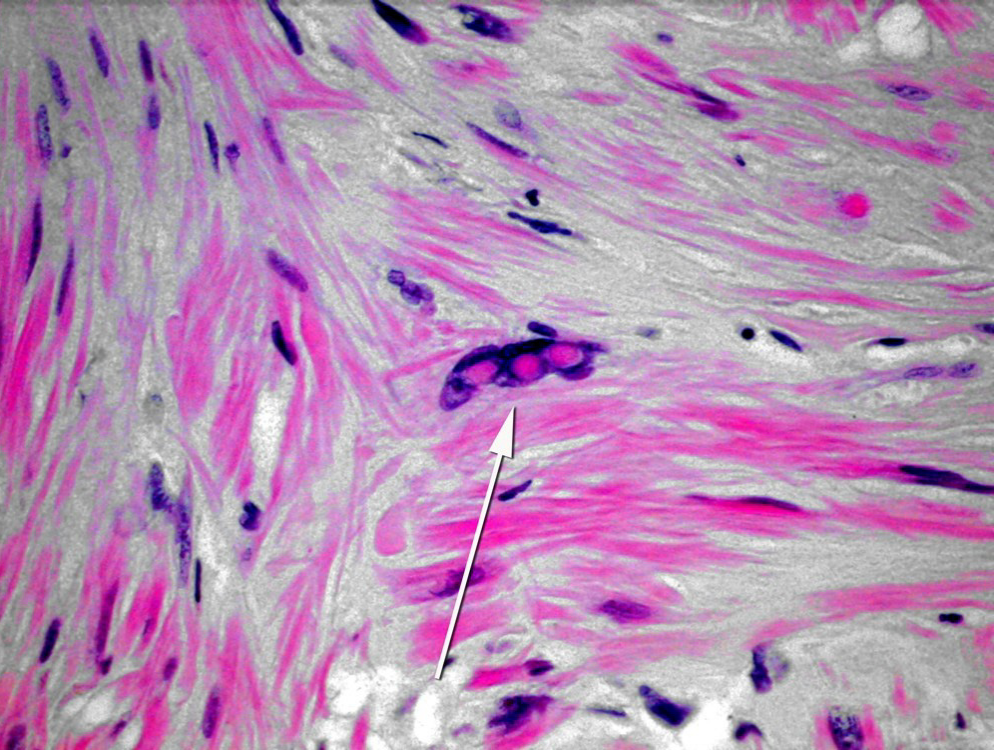
**A histopathological slide demonstrating the characteristic interlacing bundles of spindle shaped muscle cells with pleomorphic nuclei and occasional nuclear inclusions found in symplastic leiomyomas**.

On gross sections these tumours appear white-grey and are well circumscribed and encapsulated. On high power images these neoplasms are characterised by interlacing bundles of spindle shaped muscle cells with pleomorphic nuclei and occasional nuclear inclusions (Figure 2). The muscular nature of these lesions can be demonstrated by positivity for Masson-trichrome staining[5]. There are no mitotic figures seen (Figure 2). Immunohistochemically, the tumour cells express vimentin, desmin, smooth muscle actin, and muscle specific actin, but not cytokeratin, neurofilament, or glial fibrillary acidic protein[11].

It is important to emphasize that despite their histological characteristics on presentation, compatible with malignancy, these tumours have a benign course not any different from conventional leiomyomas even when they are larger in size than those reported in the literature. In contrast to scrotal leiomyosarcomas, scrotal leiomyomas with bizarre nuclei are not hypercellular, and they lack mitotic activity[11].

Ultrasound scan appears to be the investigation of choice in the pre-operative assessment of these tumours [7,9] and should ensure a proper surgical approach with simple excision of the tumour.

## Conclusion

This case report highlights some important diagnostic and treatment issues related to this rare tumour. Histologically they behave differently to both conventional leiomyomas as well as leiomyosarcomas. Their behaviour is benign in nature although it is not clear if they can recur locally. Hence follow-up of these patients is advised. This report highlights the clinicopathological characteristics of the scrotal bizarre leiomyoma in order to increase our understanding, and avoid the possibility of erroneous diagnosis and treatment. It is very important to distinguish these bizarre or symplastic leiomyomas from leiomyosarcomas to avoid unnecessary treatment.

## Consent

Written consent was obtained from the patient to publish this manuscript and accompanying images. A copy of this consent is available for review by the editor of this journal.

## Competing interests

The authors declare that they have no competing interests.

## Authors' contributions

JM and SV did the literature search and wrote the draft article. PA was responsible for the histology slide and histological input. TWC revised the article for intellectual content.
